# Efficacy of chlorhexidine bathing for reducing healthcare associated bloodstream infections: a meta-analysis

**DOI:** 10.1186/s13613-015-0073-9

**Published:** 2015-10-07

**Authors:** Eun Young Choi, Dong-Ah Park, Hyun Jung Kim, Jinkyeong Park

**Affiliations:** Department of Pulmonary and Critical Care Medicine, Yeungnam University College of Medicine, Daegu, Republic of Korea; Office of Health Technology Evaluation, National Evidence-Based Healthcare Collaborating Agency, Seoul, Republic of Korea; Institute for Evidence-based Medicine, The Korean Branch of Australasian Cochrane Center, Seoul, Republic of Korea; Department of Preventive Medicine, College of Medicine, Korea University, Seoul, Republic of Korea; Department of Critical Care Medicine in Samsung Medical Center, Sungkyunkwan University School of Medicine, # 50, Irwon-Dong, Gangnam-gu, Seoul, Republic of Korea

**Keywords:** Chlorhexidine, Mupirocin, MRSA, Critically ill, Meta-analysis

## Abstract

**Background:**

We performed a meta-analysis of randomized controlled trials (RCTs) to determine if daily bathing with chlorhexidine decreased hospital-acquired BSIs in critically ill patients.

**Methods:**

We searched the MEDLINE, EMBASE, and Cochrane Central Register of Controlled Trials databases to identify randomized controlled trials that compared daily bathing with chlorhexidine and a control in critically ill patients.

**Results:**

This meta-analysis included five RCTs. The overall incidence of measured hospital-acquired BSIs was significantly lower in the chlorhexidine group compared to the controls 0.69 (95 % CI 0.55–0.85; *P* < 0.001; *I*^2^ = 57.7 %). Gram-positive-induced (RR = 0.49, 95 % CI 0.41–0.58; *P* = 0.000; *I*^2^ = 0.0 %) bacteremias were significantly less common in the chlorhexidine group. The incidence of MRSA bacteremias (RR 0.63; 95 % CI 0.44–0.91; *P* = 0.006; *I*^2^ = 30.3 %) was significantly lower among patients who received mupirocin in addition to chlorhexidine bathing than among those who did not routinely receive mupirocin.

**Conclusions:**

Daily bathing with chlorhexidine may be effective to reduce the incidence of hospital-acquired BSIs. However, chlorhexidine bathing alone may be of limited utility in reduction of MRSA bacteremia; intranasal mupirocin may also be required. This meta-analysis has several limitations. Future large-scale international multicenter studies are needed.

## Background

Up to 20–30 % of patients admitted to intensive care units (ICUs) develop a hospital-acquired infection during their ICU stay [1]. Many of these infections are caused by multidrug-resistant organisms, such as methicillin-resistant *Staphylococcus aureus* (MRSA) and vancomycin-resistant Enterococcus (VRE), limiting the number of antibiotics available for treatment. These infections prolong the length of stay and increase the costs of care and patient morbidity and mortality [2, 3]. Center for Disease Control and Prevention (CDC) recommends hand washing and isolation for precautions, but these strategies are not easy to achieve the target. Because a lots of healthcare persons should be consistent adherence to strategies and continuously sustain [4].

Hospital-acquired infections are preceded by colonization with pathogenic bacteria, and hospital-acquired bloodstream infections (BSIs) often result from the ingress of skin organisms into the bloodstream along vascular catheters or other breaks in skin integrity [5]. Successful efforts to decolonize patients have reduced the rates of these infections. Chlorhexidine is a water-soluble antiseptic preparation with broad activity against Gram-positive and Gram-negative organisms, facultative anaerobes, aerobes, and yeasts [6]. Recent investigations of whole-body skin decolonization with chlorhexidine in critically ill patients have demonstrated reductions in the rates of VRE, MRSA, and *Acinetobacter baumannii* colonization, and an overall decrease in the incidence of central catheter-associated BSIs [7–10]. A previous meta-analysis of non-randomized controlled trials (RCTs) suggested that the practice of daily bathing with chlorhexidine decreased hospital-acquired BSIs [11]. Subsequently, some RCTs of daily bathing with chlorhexidine in the ICU have appeared [12–14].

Therefore, we conducted a meta-analysis of RCTs to determine whether daily bathing of critically ill patients with chlorhexidine decreases hospital-acquired BSIs compared to patients who received routine bathing.

## Methods and statistics

The methods for including articles and analysis and reporting the results of meta-analyses are specified a priori in a protocol developed based on recommendations in the Preferred Reporting Items for Systematic Reviews and Meta-Analyses (PRISMA) statement [15]. An ethics review of systematic reviews and meta-analysis studies, such as this study, was not required per our institutional Health Research Ethics Board.

### Literature search strategy

We searched the databases of MEDLINE (1948 to August 2014), EMBASE (1980 to August 2014), and the Cochrane Register of Controlled Trials (CENTRAL) of the Cochrane Library (Issue 8, 2014) using the search filter in the Ovid database (SIGN; http://www.sign.ac.uk). The search terms were “critical illness”, “intensive care units”, “burn units”, “coronary care units”, “respiratory care units”, “intensive care”, “ICU”, “infection control”, “universal precautions”, “decontamination”, “surveillance”, “screening”, “antisepsis”, “decolonization”, “chlorhexidine”, “Tubulicid”, and “Sebidin”. We also reviewed the bibliographies of relevant review articles to identify additional publications, and searched an international database (http://www.clinicaltrial.gov) to identify relevant ongoing or recently completed clinical trials. The search was performed without restriction with respect to language or year of publication. The last date on which a search was conducted was February 18, 2015.

### Selection criteria for studies

Two authors (JP and EYC) independently evaluated the eligibility of all studies to determine whether they met each inclusion criterion. Disagreements between the two evaluators were resolved by discussion and consensus, and with the opinion of a third reviewer (DAP). The eligibility criteria included all of the following: (a) study design, randomized controlled trials; (b) population, adult (>18 years old) critically ill patients in the ICU; (c) intervention, comparison between daily bathing with chlorhexidine and a control (daily bathing with soap and water or non-antimicrobial washcloths); and (d) outcomes. The primary outcome was hospital-acquired BSIs, defined as bloodstream infections detected more than 48 h after admission to the unit. The secondary outcomes were the types of reported microorganisms that caused hospital-acquired BSIs detected more than 48 h after admission to the unit and adverse effects of daily bathing with chlorhexidine. Studies that did not provide quantitative data for the meta-analysis were excluded.

### Data extraction and quality assessment

Two authors (JP and EYC) independently extracted the data using a standardized form. Only published data were used. The two extractors assessed the quality of the included trials using Cochrane Collaboration’s tool, and evaluated the risk of bias in randomized trials, which covers selection, performance, detection, attrition, and reporting bias [16]. High quality was defined as satisfying at least six of the seven criteria. We resolved disagreements about data extraction and quality assessment by consensus or by discussion with a third reviewer (DAP).

### Statistical analysis

**Fig. 1:**
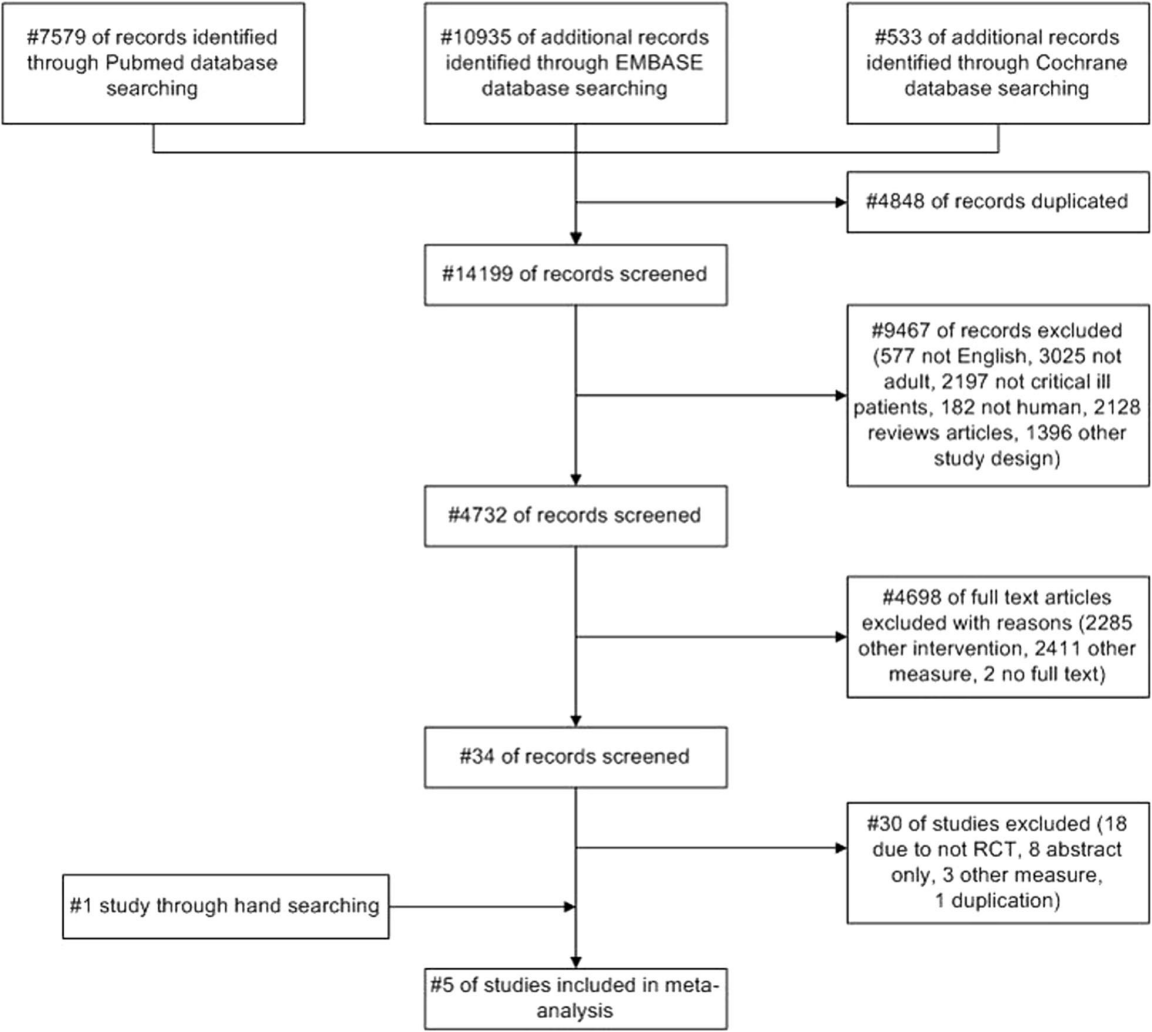
Flow-diagram of the selection criteria. Flow chart explaining the selection of eligible studies included in the meta-analysis

The clinical outcomes in our analysis can be categorized as binary or continuous data. BSIs were quantified as patient-days. One patient-day represents a unit of time during which the services of the institution or facility are used by a patient. Relative risk (RR) and 95 % confidence interval (CI) were used as the summary effect for a binary outcome, and the standardized mean difference and 95 % CI were used as the summary effect of a continuous outcome. Data were pooled using the Mantel–Haenszel method. We reported results according to a fixed-effects model in the absence of significant heterogeneity, and to a random-effects model [17] in the presence of significant heterogeneity. We used the random-effects model because it accounts for variation among studies, in addition to sampling error within studies [16]. The appropriateness of pooling data across studies was assessed using Cochrane’s *χ*^2^ test and the *I*^2^ test for heterogeneity, which measure the inconsistency across the study results and describe the proportion of the total variation in the study estimates that is due to heterogeneity, rather than sampling error. Statistically significant heterogeneity was considered to be present when *P* < 0.10 and *I*^2^ > 50 % [18]. We checked the publication bias as subgroup analysis based on differences in design, type of control, the number of study sample, concomitant using drug, and so on. We followed the guidelines of the Cochrane Handbook for meta-analysis of randomized controlled studies, and PRISMA criteria were used to evaluate research methodology (Fig. 1). Two-sided *P* values less than 0.05 were considered statistically significant. Meta-analyses, forest plots, and publication bias analyses were produced using Stata SE 13.1 for Mac (Stata Corp, TX, USA) [19]. We pre-specified the subgroup analysis according to more similar interventions or control groups.

## Results

### Literature search and study selection

Figure 1 shows the flow diagram used for study selection. We identified 18,843 citations from electronic databases, and selected 34 potentially relevant publications for a full text assessment. Of these 34 articles, 30 were excluded from this meta-analysis for the following reasons: 18 trials were not randomized controlled trials; 8 trials were only abstracts; and 3 trials [20–22] measured other outcome variables. Two trials were duplicates; we included the most recent trial only [23]. Additionally, we found one study through hand searching under writing a manuscript. Consequently, we included five studies in the final analysis [12–14, 24, 25].

### Characteristics of the included studies

All of the trials were prospective, randomized trials, and three of them were crossover trials. One trial was post hoc analyzed after the completion of randomized control. The characteristics of the studies are presented in Table 1. Four studies used cloths impregnated with 2 % chlorhexidine (the equivalent of 500 mg chlorhexidine per cloth) (Sage Products) for decolonization, and a non-antiseptic liquid soap was applied as a comparator; and one trial compared 4 % chlorhexidine (Hibiscrub; AstraZeneca, Rueil-Malmaison, France) and non-antiseptic liquid soap. The study size ranged from 2210 to 10,603 patient-days. All of the studies examined adults. A funnel plot for publication bias could not be performed because there were too few trials to analyze with the Egger test.

**Table 1:** Characteristics of the included studies

Study	Bleasdale et al. [24]	Climo et al. [12]	Huang et al. [13]	Camus et al. [25]	Noto et al. [16]
Study design	Prospective, 2-arm, crossover	Multicenter, cluster-randomized, non-blinded crossover trial	Cluster-randomized trial	Multicenter, placebo-controlled, randomized, double-blind study	Multicenter, pragmatic cluster randomized, crossover, controlled study
Location	Single center, USA	7 hospitals, USA	43 hospitals, USA	3 hospitals, France	Single center, USA
Study site	Two 11-bed medical ICUs	Eight ICUs, one BMT unit	74 ICUs	Medical ICUs	5 adult ICUs
Study period	Jun 8 to Dec 20, 2005 and Jan 5 to Jun 21, 2006	Aug 2007 to Feb 2009	Apr 8, 2010 to Sep 30, 2011	Apr 1996 to Jun 1999	Jul 2012 to Jul 2013
Intervention	Daily bathing with 2 % chlorhexidine-impregnated cloth	Daily bathing with 2 % chlorhexidine-impregnated cloth	Daily bathing with 2 % chlorhexidine-impregnated cloth and twice-daily intranasal mupirocin	Daily bathing with 4 % chlorhexidine soap and daily intranasal mupirocin	Daily bathing with 2 % chlorhexidine-impregnated cloth
Comparator	Daily bathing with soap and water	Daily bathing with non-antimicrobial washcloths	Screening and isolation for MRSA colonization	Daily bathing with non-antiseptic liquid soap	Daily bathing with disposable non-antimicrobial cloths
Number of patients					
Intervention	391	3970	26,024	259	4852
Comparator	445	3842	23,480	256	4488
Number of patient-days					
Intervention	2210	24,902	101,603	3963	20,720.5
Comparator	2219	24,983	88,222	4276	19,201.5
Other HCAI prevention	The MICU catheter insertion policy	Contact precautions	Contact precaution policies, based on the Centers for Disease Control and Prevention	Standard precautions according to the French recommendation	Contact precautions according to the usual practice of each unit
The period under observation	MICU stay	Between the control period and the intervention period	From the third day after ICU admission through the second day after ICU discharge	Between the randomization and the termination date of study treatments plus an additional 48 h	During the chlorhexidine bathing period

*BMT* bone marrow transplantation, *HCAI* healthcare associated infection, *MICU* medical intensive care unit, *MRSA* methicillin resistant *S. aureus*

### Risk of bias in the included studies

Our assessments of each risk of bias item for each randomized controlled study are summarized in Table 2. Three [12–14] of five trials were quasi-experimental, with limited to no assessment of potential confounding factors. Four studies were cluster-randomized trials. We assessed these studies as being at low risk of allocation concealment. We assessed the study as being high risk of having a funding-related item when there were grants or support from a company. However, when public funding was used, we assessed the studies as low-risk.

**Table 2:** Risk of bias assessment for the randomized controlled studies included in this meta-analysis

Study	Bleasdale et al. [24]	Climo et al. [12]	Huang et al. [13]	Camus et al. [25]	Noto et al. [16]
Adequate sequence generation?	Low	Unclear	Low	Unclear	Low
Allocation concealment?	Low	Low	Low	Low	Low
Blinding of participants and personnel?	High	High	Unclear	Low	High
Blinding of outcome assessment?	Low	High	Low	Low	Low
Incomplete outcome data addressed?	Low	Low	Low	Low	Low
Free of selective reporting?	Low	Low	Low	Low	Low
Free of potential bias relevant industrial funding?	High	High	Low	High	Low

### Primary outcome: all-cause hospital-acquired BSIs

**Fig. 2:**
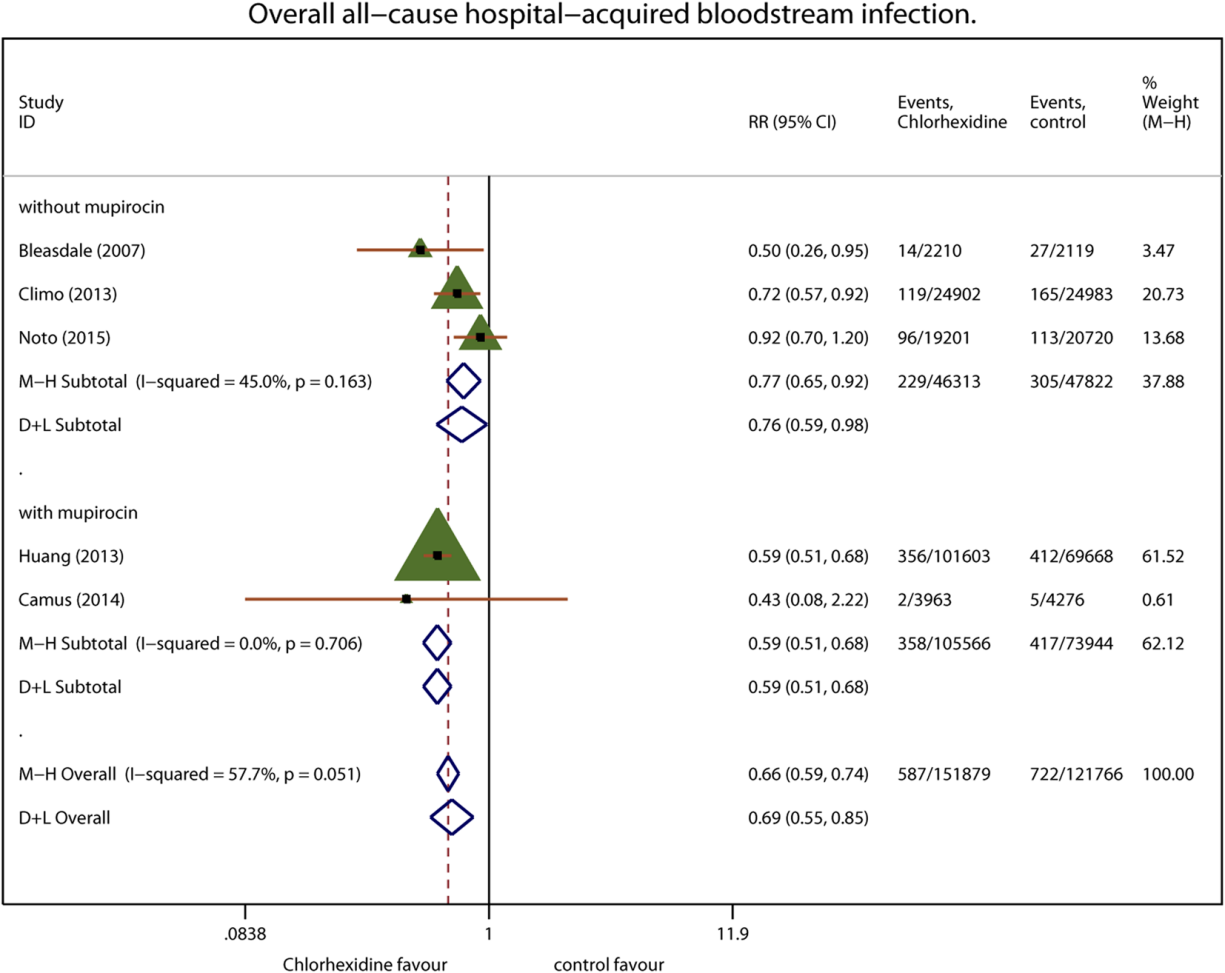
The overall incidence of hospital-acquired bloodstream infections. Each effect size is shown with its confidence interval (CI) as *solid triangle*. The overall effect and CI are shown as a *diamond* with a *dotted line* indicating its location. *Vertical solid line* at *1* indicates no treatment effect. *M–H* Mantel–Haenszel weighted fixed effects, *D* *+* *L* random-effects estimate

The primary outcome was the overall incidence of measured hospital-acquired BSI; 587 BSI events developed in the chlorhexidine group over 151,879 patient-days, compared to 670 in the control arm over 140,320 patient-days. Fixed-effects modeling yielded an RR of 0.82 (95 % CI 0.73–0.91; *P* < 0.001; *I*^2^ = 20.6 %). Figure 2 summarizes the primary outcome.

### Exploring the heterogeneity

Subgroup analyses of the potential effect of the concomitant use of mupirocin were performed to determine the effects on measured and reported hospital-acquired BSI. Subgroup analysis at the results for hospital-acquired BSI was more homogeneous. Subgroup analysis featuring the concomitant use of intranasal mupirocin yielded a pooled RR of 0.59 (95 % CI 0.51–0.68; *P* < 0.001; *I*^2^ = 0.0 %). Subgroup analysis classified by the type of control group determined that the pooled RR was 0.68 (95 % CI 0.55–0.85; *P* = 0.001; *I*^2^ = 0.0 %) between the groups washed with non-antimicrobial soap to treat hospital-acquired BSI. A subgroup analysis classified by the concentration of chlorhexidine revealed no significant difference in hospital-acquired BSI. The pooled RR was 0.82 (95 % CI 0.73–0.92; *P* < 0.001; *I*^2^ = 32.5 %) between 2 % chlorhexidine groups. The pooled RR for reported hospital-acquired BSI was 0.82 (95 % CI 0.73–0.92; *P* < 0.001; *I*^2^ = 32.5 %) when a study performed as post hoc analysis in the 1990s was excluded. The pooled RR for reported hospital-acquired BSI was 0.77 (95 % CI 0.65–0.91; *P* = 0.002; *I*^2^ = 27.1 %) when a largest study was excluded.

Of all hospital-acquired BSIs, central catheter-related BSIs were defined as BSIs noted in patients for whom at least one central venous catheter was placed within 48 h before detection of the infection. Two studies presented quantitative data [12, 14]; 30 central catheter-related BSI events developed in the chlorhexidine group over 14,824 catheter-days, compared to 65 in the control arm over 14,297 catheter-days. Fixed-effects modeling yielded an RR of 0.44 (95 % CI 0.28–0.67; *P* < 0.001; *I*^2^ = 0.0 %).

### Secondary outcomes

#### Microorganisms were isolated from bloodstream infections

In total, 475 microorganisms for 132,678 patient-days in the chlorhexidine group and 543 microorganisms for 119,600 patient-days in the control group were isolated in BSIs (RR 0.73, 95 % CI 0.57–0.93; *P* = 0.001, *I*^2^ = 51.1 %).

**Fig. 3:**
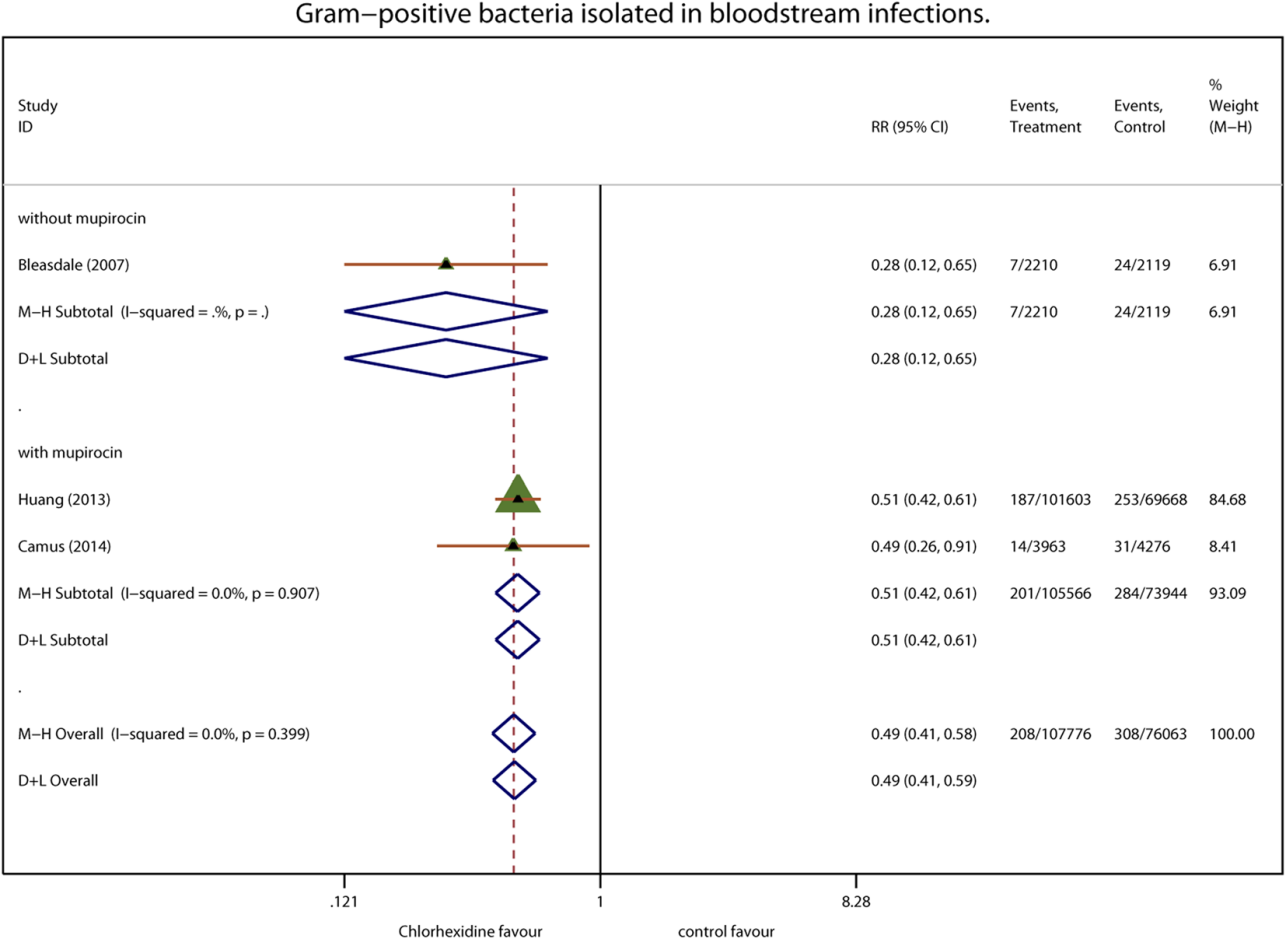
Gram-positive bacteria isolated from bloodstream infections. Each effect size is shown with its confidence interval (CI) as *solid triangle*. The overall effect and CI are shown as a *diamond* with a *dotted line* indicating its location. *Vertical solid line* at *1* indicates no treatment effect. *M–H* Mantel–Haenszel weighted fixed effects, *D* *+* *L* random-effects estimate

**Fig. 4:**
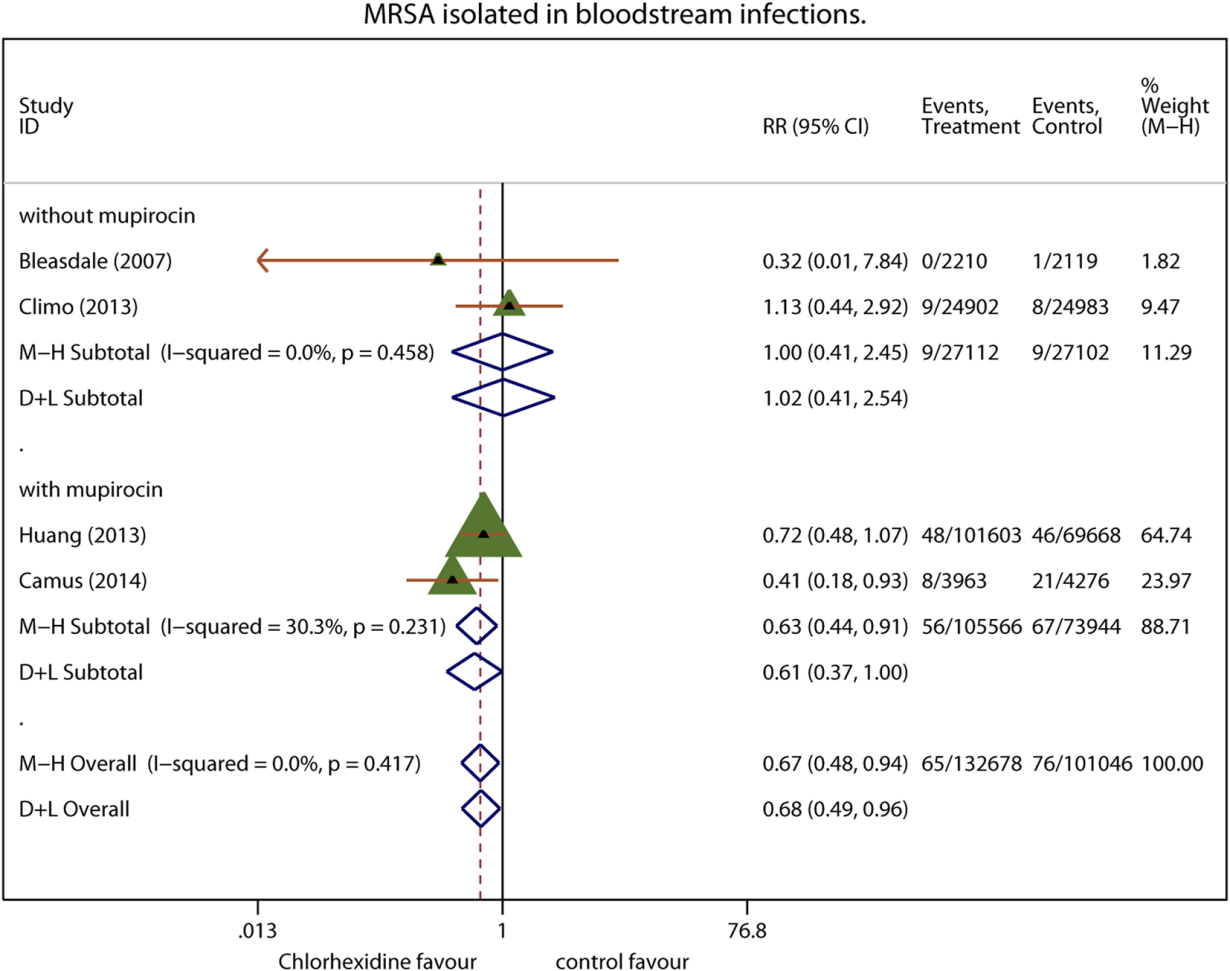
Methicillin resistant *S. aureus* isolated from bloodstream infections. Each effect size is shown with its confidence interval (CI) as *solid triangle*. The overall effect and CI are shown as a *diamond* with a *dotted line* indicating its location. *Vertical solid line* at *1* indicates no treatment effect. *M–H* Mantel–Haenszel weighted fixed effects, *D* *+* *L* random-effects estimate

Four of the five trials in this meta-analysis reported the isolation of Gram-positive pathogens. Overall BSIs caused by Gram-positive pathogens involved 251 events in 132,678 patient-days with chlorhexidine compared to 351 events for 119,600 patient-days in the controls. Figure 3 summarizes the Gram-positive pathogens isolated. The summary effect of Gram-positive pathogens had a pooled RR of 0.59 (95 % CI 0.44–0.79; *P* < 0.001; *I*^2^ = 46.0 %) in a random-effects model. Subgroup analysis yielded more homogeneous results for Gram-positive pathogen-related BSIs. Subgroup analysis of mupirocin use in conjunction with chlorhexidine bathing yielded a pooled RR of 0.69 (95 % CI 0.57–0.83; *P* = 0.001; *I*^2^ = 21.6 %). There were significantly fewer MRSA-related BSIs with chlorhexidine than in the controls (pooled RR 0.64; 95 % CI 0.47–0.88; *P* = 0.006; *I*^2^ = 0.0 %; Fig. 4). In subgroup analysis by mupirocin use, MRSA-related BSIs were significantly fewer in the group featuring concomitant use of intranasal mupirocin and chlorhexidine bathing the chlorhexidine bathing compared to chlorhexidine bathing alone (pooled RR 0.63; 95 % CI 0.44–0.91; *P* = 0.013; *I*^2^ = 30.0 %).

Three of the five trials indicated the isolation of Gram-negative pathogens. The infections caused by Gram-negative pathogens involved 132 of 32,204 patient-days with chlorhexidine compared to 111 of 29,441 patient-days in the controls. The summary effect of Gram-negative pathogens was a pooled RR of 1.09 (95 % CI 0.85–1.40; *P* = 0.51; *I*^2^ = 0.00 %) in the fixed-effects model.

Three of the five trials isolated fungal pathogens. There were 73 fungal infections for 32,204 patient-days with chlorhexidine compared to 70 for 29,441 patient-days in the controls. The summary effect of fungal infection was a pooled RR of 0.83 (95 % CI 0.42–1.62; *P* = 0.56; *I*^2^ = 52.1 %) in the random-effects model.

#### Adverse effects: skin rash

Four of the five studies included in this meta-analysis reported chlorhexidine-related skin rashes as an adverse effect of chlorhexidine. In total, 92 events in the chlorhexidine group developed over 132,678 patient-days compared to 136 events in the control arm over 119,600 patient-days. A random-effects model resulted in an RR of 1.20 (95 % CI 0.43–3.31; *P* = 0.73; *I*^2^ = 56.3 %).

## Discussion

In this meta-analysis of five randomized controlled trials, we found that daily bathing with chlorhexidine reduced the development of hospital-acquired BSIs more effectively with the concomitant use of intranasal mupirocin. BSIs caused by Gram-positive cocci in critically ill patients also decreased significantly. However, chlorhexidine bathing had a limited effect on reducing BSIs caused by MRSA. MRSA-BSIs were significantly fewer in only subgroups in the concomitant use of intranasal mupirocin. And, chlorhexidine bathing would be helpful to reduce the incidence of Gram-negative bacteremia and fungemia. The overall incidence of adverse events such as skin rashes was similar between daily bathing with chlorhexidine and the control.

Our data were consistent with those in the meta-analysis reported by O’Horo et al. [11] or Derde et al. [26] which indicated efficacy of daily bathing with chlorhexidine in order to decrease hospital-acquired BSIs. O’Horo et al. included only 1 RCT [24] and 11 observational studies. They pooled the studies together regardless of study design. We included five RCTs and removed observational trials. As the previous report, chlorhexidine bathing reduced the development of hospital-acquired BSIs caused by Gram-positive cocci. In recent evidence [11, 26] (not from RCTs) also reported that chlorhexidine bathing effectively prevented MRSA BSIs in critically ill patients. These studies did not distinguish whether effects came from chlorhexidine alone or combination of mupirocin and chlorhexidine. Our results showed some limitations about chlorhexidine bathing alone. In enrolled studies in our meta-analysis, the combination group had studies with higher quality and larger than chlorhexidine alone group. One study in combination group completed more than 15 years ago (1996–1999), several practices that may impact catheter-related BSIs have changed significantly during those years. Prevention of MRSA seems to require a combination of chlorhexidine and mupirocin. In our results, daily bathing with chlorhexidine did not affect BSI caused by Gram-negative bacteria or fungi. This may be because Gram-negative BSIs often originate from the lung or digestive tract, and are therefore not impacted by the chlorhexidine skin wash.

Our meta-analysis had some limitations. First, it involved a small number of studies, and the five RCTs in this meta-analysis had various study designs: one of five trials was a 2 × 2 factorial design [25] with other interventions (topical polymyxin and tobramycin) and post hoc analysis. Three trials [12–14] of five trials were crossover or cluster random designs. It may be considerably smaller number of RCT studies when it is assumed that a cluster enrollment. Infection rates are different in each ICU. The infection related interventions could be affected by the infection state neighbor patients. Maybe even given these limitations are infection-related design seems to be a cluster randomized controlled trials possess a greater advantage. We tried to overcome these limitations according to various study design with multiple sensitivity-test. However, our results were continuously constant.

Second, most of the studies enrolled in this meta-analysis were not high quality due to the open-label study design. Additionally, three of the five studies could not guarantee blinded studies. The studies for infection were strikingly influenced by adherence to infection control. It was possible to overestimate the intervention effects. Therefore, caution is needed when interpreting the results. Third, the microorganisms reported were very different. One study [25] reported only *S. aureus* bloodstream infections. For fungi, two studies [13, 24] reported only *Candida*, one study [12] reported *Candida* and others, and the other did not report fungi. The results for Gram-negative bacteria came from three of the five trials. This might contribute to a lack of confidence in the results. Fourth, none of studies reported the baseline patient characteristics. We only estimated the similarity between studies or groups based on a low *I*^2^ value. Fifth, one [25] of the five studies used 4 % Hibiscrub^®^ soap instead of 2 % chlorhexidine cloths. The chlorhexidine of the soap might have been diluted; lower concentrations of chlorhexidine exert bacteriostatic effects, thus being less effective than chlorhexidine-impregnated cloth bath. To overcome this problem, we performed a subgroup analysis by chlorhexidine concentration, and found no significant difference in hospital-acquired BSIs, including those caused by Gram-positive bacteria or MRSA. Sixth, four of the five studies were performed in the United States and the other was performed in France; thus, the regional environment in the United States could have affected these results. A multinational RCT on this topic is necessary to overcome these limitations.

In conclusion, daily bathing with chlorhexidine was associated with reductions in the rates of measured hospital-acquired BSI without significant complications in critically ill patients. Daily bathing with chlorhexidine decreased the incidence of Gram-positive bacteremia regardless of mupirocin use. However, chlorhexidine-only bathing may not be entirely effective to decrease MRSA-related hospital-acquired BSIs. We should consider to emergence of resistance when daily chlorhexidine bathing in the ICUs was implemented [27]. Further multinational, multicenter RCTs are required to overcome the limitations of the meta-analysis.
